# Nasal Polyposis: Insights in Epithelial-Mesenchymal Transition and Differentiation of Polyp Mesenchymal Stem Cells

**DOI:** 10.3390/ijms21186878

**Published:** 2020-09-19

**Authors:** Emanuela Chiarella, Nicola Lombardo, Nadia Lobello, Annamaria Aloisio, Teodoro Aragona, Corrado Pelaia, Stefania Scicchitano, Heather Mandy Bond, Maria Mesuraca

**Affiliations:** 1 Laboratory of Molecular Haematopoiesis and Stem Cell Biology, Department of Experimental and Clinical Medicine, University “Magna Græcia”, 88100 Catanzaro, Italy; emanuelachiarella@unicz.it (E.C.); aloisio@unicz.it (A.A.); scicchitano@unicz.it (S.S.); 2 Otolaryngology Head and Neck Surgery, Department Medical and Surgical Sciences, University “Magna Græcia”, 88100 Catanzaro, Italy; nlombardo@unicz.it (N.L.); nadialobello@gmail.com (N.L.); 3 Otolaryngology, A.O.U. Ospedali Riuniti, 60123 Ancona, Italy; teodoroaragona@yahoo.it; 4 Department of Health Sciences, University “Magna Græcia” of Catanzaro, 88100 Catanzaro, Italy; pelaia.corrado@gmail.com

**Keywords:** chronic rhinosinusitis (CR), inflammation, nasal polyps, epithelial to mesenchymal transition (EMT), Nasal polyp derived mesenchymal stem cells (PO-MSCs)

## Abstract

Chronic rhinosinusitis is a common inflammatory disease of paranasal sinuses, which causes rhinorrhea, nasal congestion, and hyposmia. The genetic predisposition or the exposure to irritants can sustain the inflammatory response and the development of nasal polyposis. Nasal polyps are benign and teardrop-shaped growths that project in the nasal cavities, and originate from the ethmoid sinuses. This inflammatory process is associated with high expression of IL-4, IL-5 and IL-13 and IgE. Antibodies targeting these cytokines or receptors represent a therapeutic strategy in the treatment of nasal polyposis in combination with corticosteroids. The molecular pathogenesis of nasal polyps in chronic rhinosinusitis (CRS) patients is associated with remodeling transition, a process in which epithelial cells lose their typical phenotype, acquiring a mesenchymal-like aspect. TGFβ/SMAD, ERK, and Wnt/β-catenin pathways are altered during the nasal tissue remodeling. miRNA and inhibitor molecules targeting these signaling pathways are able to interfere with the process; which could lead to alternative therapies. Nasal polyps are an alternative source of mesenchymal stem cells, which can be isolated from surgical biopsies. A molecular understanding of the biology of PO-MSCs will contribute to the delineating inflammatory process underlying the development of nasal polyps.

## 1. Introduction

### 1.1. Chronic Rhinosinusitis (CRS) Classification

The nasal disorder of CRS is a chronic condition of the upper airway characterized histologically by the infiltration of inflammatory cells like eosinophils or neutrophils of the paranasal sinuses and nasal cavity [1,2] affecting 5–12% of the population. Clinically, nasal polyposis have nasal obstruction, hyposmia, rhinorrhea and reduced quality of life. Nasal polyps (NP) derived from the middle meatus are inflammatory outgrowths of paranasal sinus mucosa, most often benign, frequently bilateral, and typically develop in adulthood, and are characterized by inflammation [3,4]. They present an abnormal remodeling response and a lack of immunoregulation, creating an imbalance and, consequently, favoring inflammation.

According to the European Position Paper on Rhinosinusitus and Nasal Polyps (EPOS ) 2012 guidelines, CRS is defined as inflammation of the nose and paranasal sinuses characterized by the presence of two or more of the following symptoms for greater than 12 weeks duration: nasal blockage/obstruction/congestion; nasal discharge; facial pain/pressure; reduction or loss of smell. Confirmation of the diagnosis of CRS with nasal polyps (CRSwNP) or CRS without nasal polyps (CRSsNP) is made by nasal endoscopy or sinus CT scan. In adults, nasal polyps seen in both nasal passages and any unilateral polyps should be concerning for an alternative etiology such as malignancy. The endotype complement of a CRS patient is defined by a set of interleukins (ILs), cytokines, growth-factors, and immunological inducers. Clusters are identified based on analysis of IL-5, IFN-γ, IL-17A, TNF-α, IL-22, IL-1β, IL-6, IL-8, cationic eosinophilic protein, myeloperoxidase, TGF-β1, IgE, staphylococcus aureus-specific IgE for enterotoxin, and albumin in CRS in the nose itself and are highly modified with the disease [2,5]. It is now evident that this classification is insufficient, since the same patients may or may not have nasal polyps at different times in their clinical history, even after appropriate medical or surgical treatment [5].

Recently, according to the EPOS 2020 guidelines [3], CRS can be classified into primary or secondary disease based on the extent of anatomical involvement and endotype dominance. With localized primary CRS, which are usually unilateral or bilateral in distribution, those with typically inflammatory endotype dominance are separated into a type 2 skewed inflammation and can be exacerbated in allergic fungal rhinosinusitis (AFRS, ) while non-type 2 disease is found in isolated sinusitis. Instead diffuse (bilateral) primary CRS type 2 can result in a phenotype associated with CRSwNP/eCRS (eosinophilic CRS), AFRS or CCAD (central compartment allergic disease). Non-eCRS is a characteristic phenotype of diffuse primary CRS type 2. Additionally, secondary CRS can be classified into localized or diffuse according to the anatomic distribution. In the first case, the endotype dominance is attributable to local pathology, in the second case, it is dependent on mechanical, inflammatory, and immunological factors. The secondary CRS involves a wide range of clinical phenotypes including fungal ball, tumors, primary ciliary dyskinesia, cystic fibrosis, granulomatosis with polyangiitis (GPA) (Wegener’s disease), and eosinophilic granulomatosis with polyangiitis (EGPA) (Churg-Strauss disease) with different immunodeficiency’s [1,3].

### 1.2. Etiology of Polyp Formation

Nasal polyps are non-cancerous and painless growths originating from the ethmoid sinuses and affect the nasal mucosa and paranasal sinuses. The etiology of polyps formation is not yet fully understood [6,7]. Numerous factors including anatomic disorders, genetic factors, infections caused by viruses, bacteria, fungi, as well as asthma, allergic rhinitis, non-allergic inhalants are associated with nasal polyp development and progression [8]. The nasal polyp formation involve histologically-identified features: the mucosal epithelial rupture, proliferation of fibrous tissue through the injured epithelial, extracellular matrix (ECM) accumulation with edema, and the proliferation of a granular tissue comprising thin-walled vessels and inflammatory cell infiltration [9,10]. The inflamed nasal mucosa takes on a gelatinous texture, resembling grape like clusters, with a translucent and pale pink appearance, giving rise to nasal polyps [11].

### 1.3. Inflammation in Nasal CRS

The inflammatory process plays an important role in the pathogenesis of nasal polyposis. The different forms of CRS appear to be caused by inflammatory changes in the sinonasal mucosa. A Type 2 T helpers (Th2)-mediated inflammatory process [4] is usually found in CRSwNP, whereas both Th2- and Th1-mediated processes are found in CRSsNP.

CRSwNP can have different inflammatory profiles depending on related diseases, such as bronchial asthma, cystic fibrosis (CF), or NASID-exacerbated respiratory disease (N-ERD). The inflammatory process is characterized by a multitude of cytokines and interleukins in the different cell types, importantly IL-4, IL-5, and IL-13, which are produced by Th2 cells.

Comparing Th2/Th1/Th17 cytokine patterns and markers of eosinophilic and neutrophilic inflammation in patients with CRS from six regions covering Europe, Asia, and Australia [12], higher levels of Th2 and IL-5, together to eosinophilic inflammation, are found in European patients with CRSwNP; on the other hand, neutrophilic inflammation combined with Th1/Th17 endotypes were found in southern Chinese patients. In addition, patients with CRSsNP from central China have a Th1-predominant pattern comparable to European patients with CRSsNP. Asian patients with eosinophilic CRSsNP showed a Th2-biased inflammation accompanied also by Th17 reactions. These population variations may mask genetic differences, such that a specific gene/s associated have yet not been found [12].

Nasal polyp tissue (CRSwNP) displays robust levels of the cytokines IL-4, IL-5, and IL-13 compared to healthy controls, suggesting that they are key regulatory factors for eosinophil survival and activity. IL-4 is able to promote the differentiation of CD4^+^ T cells into Th2 cells and at the same time inhibit interferon (IFN)-γ production and the Th1 response. IL-5 produced by Th2 [13] as well as type 2 innate helper lymphoid cells (ILC2) [14], induces proliferation and maturation of eosinophils and is essential for their mobilization. Subsequently eosinophils migrate and accumulate into nasal tissues where they synthesize and release lipid mediators and enzymes causing edema and tissue damage, respectively [15]. IL-13 functions as an effector molecule that mediates eosinophilic inflammation, airway hyper-responsiveness, and mucus hypersecretion [13]. IL-13 in nasal polyps type 2 acts by binding the IL-4R, activating the IRS1/2 signaling and STAT6 cascade [16].

### 1.4. Immunological-Based Therapies

Treatment protocols with saline nasal irrigation, antibiotics, topical and oral corticosteroids, and surgical removal have only limited effects in curing the disease. The revelation of the immunological mechanisms underlying chronic rhinosinusitis with nasal polyps has resulted in the production of monoclonal antibodies targeting cytokines or their receptors crucial for CRSwNP. Patients are treated with nasal sprays of corticoid steroids, mometasone furoate, and subcutaneously locally injected with monoclonal antibodies [17]. The effect of IL-5 can be antagonized by two types of humanized monoclonal antibodies targeting the IL-5 cytokine (reslizumab and mepolizumab) or the IL-5 receptor (benralizumab) [18]. Both kinds of antibodies are able to inhibit IL-5 signaling and to induce apoptosis of target cells via antibody-dependent cell-mediated cytotoxicity [19].

CRSwNP is characterized by IgE hyper production and eosinophilic inflammation. The antibody against-IgE (omalizumab) has demonstrated efficacy in patient trials with CRSwNP and comorbid asthma [20].

IL-4 and IL-13 are two key cytokines having a role in supporting CRSwNP and are expressed during type 2 inflammation, both acting through the alpha subunit of same receptor (IL4R). Dupilumab has been shown to bind to IL-4Rα, suppressing the inflammatory process dependent on both IL-4 and IL-13 signaling. IL-4 plays a role in activating fibroblasts to induce tissue remodeling, and produce an increased amount of CCL11 (eotaxin-1) [21]. IL-13 is a profibrotic cytokine, secreted from Th2 cells, associated to allergic inflammation and is involved in tissue remodeling [22], molecular mechanisms required for the transition to nasal polyp formation.

The monoclonal antibody (dupilumab) has been tested in proof-of-concept trials and scored well in comparison with anti-IL-5 (mepolizumab) and anti-IgE (omalizumab) in CRSwNP [23]. On the basis of these studies, [16] two large clinical trials at stage III [24] have now been published. Dupilumab treatment in the clinic significantly reduced polyp size, sinus opacification and severity of nasal congestion.

These biological monoclonal-based therapies are very promising clinically and were shown to be safe and well tolerated, although associated with a considerable cost. Given that the numbers of patients that suffer from CRSsNP or CRSwNP is high, such that appropriated classification is needed for patient selection. (EPOS 2020) [3].

In this review, we discuss the current knowledge of the molecular mechanisms underlying the development of nasal polyps with considering regulatory aspects regarding intra-cellular signaling. The understanding of the different cell signal transduction pathways involved in polyp pathogenesis will give the possibility to identify novel molecular-target agents that could be used to complement current therapeutic strategies. It is also highlighted that nasal tissue and polyps can be an alternative source of mesenchymal stem cells with a potential in biological co-culture therapies. Polyp-derived mesenchymal stem cells (PO-MSCs) are also useful in vitro models for studying the differentiative aspects and immune modulatory properties in the nasal polyp microenvironment.

## 2. Intra-Cellular Signaling Transduction Mechanisms Underlying the Development of Nasal Polyps (CRSwNP)

Healthy nasal epithelium consists of four cell types: basal cells, goblet cells, ciliated, and non-ciliated columnar cells [25]. Basal cells have been identified as stem/progenitor cells able to self-renew and differentiate into other epithelial cell types [26]. Stem/progenitor cells have a central role in tissue homeostasis, repair and regeneration of mucous membrane including the nasal mucosa [27]. The cellular pathogenesis of nasal polyps is related to a homeostatic imbalance between the reduction in proliferation of nasal epithelial stem/progenitor cells [28], and the presence and differentiation of mesenchymal stem/progenitor cells (MSCs) [29].

Epithelial mesenchymal transition (EMT) is part of this complex cellular process by which, epithelial cells lose their epithelial phenotype and acquire a mesenchymal one, following a chronic stimulus [30,31]. During EMT, on the one hand, epithelial markers, for example E-cadherin, are down-regulated by several inducers of EMT acting as transcription factors such as Snail, Slug, Twist, and Zeb; on the other hand, an upregulation of mesenchymal markers such as N-cadherin, alpha-smooth muscle actin (α-SMA), vimentin, and fibronectin, as well as matrix metalloproteinases (MMP) occur [32]. The process of polypogenesis results in a remodeling of the nasal tissue having a weakening of cell-to-cell contacts and increase of motility. Many factors act through different intra-cellular signaling pathways regulating polypogenesis, such as TGF-β1/SMAD3, HIF-1α, AGE/RAGE/ERK, MEK1/2-ERK1/2, Wnt/β-catenin/GSK3, and PPARγ to achieve the reorganizing of the tissue. The signaling acts in coordination with inflammatory cytokines and unbalance can result in polyp formation and sustained proliferation.

### 2.1. TGF-β1 Is Involved in CRSwNP Pathogenesis

TGF-β1-signaling dysregulation is found in inflammatory polyps where it participates to sustain the characteristic remodeling of nasal mucosa [33]. Down-regulation of TGF-β1 is typically associated with CRSwNP, whereas TGF-β1 up-regulation is characteristic of CRSsNP [34]. TGF-β1 signaling acts as a potent driver in EMT during nasal polyp formation and growth, inducing a loss of epithelial and gain of mesenchymal markers, verified by TSA, HDAC 1/2 inhibitor [35].

TGF-β1 pathway activation increases the expression of endoplasmic reticulum (ER) stress markers (XBP-1s and GRP78) [36], which are involved in inducing EMT in different cell types, such as alveolar epithelial cells and thyroid epithelial cells [37], and plays a role in fibrotic remodeling during chronic inflammatory disease. Treatment with the chemical chaperones, PBA (4-phenylbutylic acid) or PP2 (c-Src kinase inhibitor) were demonstrated to be able to block the EMT induced by TGF-β1 via the c-Src pathway in primary nasal epithelial cells (PNECs) [36].

Recent studies have demonstrated a role for miR-21 in mediating TGF-β1-induced EMT in primary human nasal epithelial cells via the PTEN/Akt pathway during the pathogenesis of CRSwNP [38]. miR-21 inhibitors could be considered as anti-polyp drugs for treating nasal polyps [38] as well as recent findings that suggest glucocorticoids might prevent tissue remodeling by blocking the EMT initiated by TGF-β1-induced MAPK and Snail/Slug signaling pathways in CRSwNP [38,39].

### 2.2. SMAD3 and HIF-1α Signaling Are Involved in CRSwNP

Epithelial cells of nasal polyps show an abnormal expression α-SMA and when they were cultivated in hypoxia conditions, EMT was induced via a SMAD3-dependent mechanism suggesting the crucial role for EMT in the pathogenesis of nasal polyps [40]. Shin et al. demonstrated that hypoxia-induced EMT independently of TGF-β1 signaling, by the suppression of PP2Ac (serine/threonine-protein phosphatase 2A catalytic subunit alpha isoform), which is the catalytic subunit of protein phosphatase 2A implied in the dephosphorylation of phospho-Smad3 [40]. In nasal epithelium, EMT is driven cooperatively by Smad3 and HIF-1α. Under hypoxia conditions, hNECs expressed HIF-1α and HIF-2α: the first one mediates cytoskeletal rearrangement during hypoxia and the loss of E-cadherin during EMT, the second protein could promote polyp growth by inducing cell proliferation. HIF-1α inhibitors such as 2ME2 (methoxyestradiol) and 17-AAG (17-allylaminogeldanamycin) were found to suppress polypoid lesion development in a murine NP model, opening the way for novel therapeutic strategies for nasal polyposis treatment [40].

### 2.3. MEK1/2-ERK1/2 Signaling Pathways in CRSwNP

The MEK1/2-ERK1/2 pathway is activated in CRSwNP [41]. There was an induction of the amount of MEK1/2 and phosphorylation of MEK1/2 and ERK1/2 in nasal polyps compared to healthy nasal mucosa. In CRSwNP patients, MEK, pMEK, and ERK were localized primarily to the cells facing the basal membrane and were scarcely in the upper layers of the epithelium and stroma. pERK was found in the nuclei of all of the cell layers in the epithelium of the polyps and was highly evident in the cells from the stroma of the turbinates of patients with CRSwNP. It appeared that ERK was activated in the epithelium of nasal polyps, associated with a role in acceleration of the cell cycle.

### 2.4. AGE/RAGE/ERK Signaling Pathways in CRSwNP

Studies have demonstrated that the AGE/RAGE/ERK pathway is involved in the pathogenesis of CRSwNP promoting EMT and tissue remodeling. The AGE/RAGE complex activated the ERK pathway sustaining trans-differentiation of epithelial cells into mesenchymal cells and facilitating stromal tissue oedema formation and tissue remodeling [42].

The interaction between the products of non-enzymatic glycation and oxidation of proteins and lipids (AGE) with the receptor of advanced glycosylation end products (RAGE) can be implied in the activation of several pathways including p38 mitogen-activated protein kinase (MAPK) and NF-κB [43] delineated in pelvic organ prolapse.

In the patients with neutrophilic chronic rhinosinusitis, the ERK pathway is typically activated by high IFN-γ expression. This activation correlated with an induction of markers of the EMT. IFN-γ promoted the EMT in human nasal epithelial cells via both the JAK-STAT1-ICSBP-p38 as well as the ERK signaling pathways. The levels of expression of p-ERK and p-p38 increased with CRS progression in an independent-manner from the hypoxia-inducible factor (HIF-1α), SMAD, and NF-κB-signaling pathways. The p38 inhibitor (SB203580) and MEK inhibitor (PD98059) were confirmed to be able to recapitulate the EMT hNECs phenotype [44]. Similarly, in a murine nasal polyp (NP) model, the number of NP lesions decreased after treatment with p38 and ERK inhibitors as well as the secretion of neutrophils but not eosinophils. The targeting of p38 and ERK signaling pathways was proposed to be a novel therapeutic strategy against neutrophil-dominant CRS [44].

### 2.5. WNT/β-Catenin/GSK Signaling Are Involved in CRSwNP

Wnt signaling dysregulation contributes to the impairment of epithelial function in CRSwNP. The up regulation of canonical Wnt signaling in CRSwNP results in an increase of β-catenin [45]. Although the mechanism is not completely elucidated, β-catenin accumulated in the cytosol moves to the nucleus where it cooperates to activate mesenchymal-related genes such as α-smooth, muscle actin, and vimentin.

The canonical WNT signaling activation by rhWNT3A or CHIR99021 (glycogen synthase kinase 3 inhibitor, a canonical Wnt agonist) treatment induced a significant increase of pro-inflammatory cytokines release in an in vitro model of normal HNEpCs define. Pro-inflammatory molecules are able to drive morphological changes in the epithelium, typical feature of remodeling in NPs [46].

Glycogen synthase kinase 3 is an important regulator of inflammatory processes involved in promoting the production of inflammatory cytokines (TNF, IL-1β, IL-6). A high expression of phosphorylated GSK-3 was detected in the nasal polyp tissue of patients with CRSwNP compared with healthy mucosa [47]. Recent studies have shown that the monoterpene oxide 1,8-cineol is able to negatively modulate the Wnt/β-catenin signaling pathway by GSK-3 dephosphorylation in nasal polyps of chronic rhinosinusitis patients [48]. The presence of Wnt, the loss of E-cadherin, and increased β-catenin are important molecular parameters that define the EMT process modulated by the Wnt inhibitors.

### 2.6. PPARγ Signaling Pathway Plays a Role in CRSwNP

PPAR-γ belongs to a superfamily of nuclear hormone receptors and has the function of modulating lipid/lipoprotein metabolism, cell cycle progression, cellular proliferation, and differentiation after binding to ligand, and has been described to be involved in EMT in CRSwNP. It has been shown that the PPAR-γ agonist rosiglitazone (ROG) has an inhibitory effect on HMGB1 (high mobility group box 1), a pro-inflammatory DNA-binding nuclear protein, inducing the epithelial cells to become mesenchymal-like cells and supporting the pathogenesis of eosinophilic chronic rhinosinusitis with nasal polyps ECRSwNP [49]. The agonist ROG reverted the effect of rhHMGB1 on EMT in ECRSwNP cells as well as the endogenous expression of HMGB1 induced by the treatment with lipopolysaccharide (LPS). ROG is able to restore the effects of HMGB1 activation up-regulating the expression of Zonula occludens-1 (ZO-1) and E-cadherin, and down-regulating the expression of N-cadherin and vimentin, biomarkers of MSCs [49].

Table 1 summarizes key pathways that are modulated after remodeling in CRSwNP.

Epithelial to mesenchymal transition is a key event in the airway remodeling, especially in the CRS endotype groups, sustained by chronic inflammatory conditions that lead to tissue remodeling. In this context, the abundant production of inflammatory molecules correlates with epithelial-mesenchymal transition such that human nasal epithelial cells lose their typical phenotype acquiring a mesenchymal one [8].

## 3. Gene Expression Studies on Nasal Polyps and Their Derived MSCs Cells

To identify the molecular properties of PO-MSCs, de Oliveira et al. [50] carried out a global gene expression profile of PO-MSCs in comparison with BM-MSCs. Comparing 4 samples of each, 15 genes were significantly upregulated including PROM1 or CD133, a stemness marker typical of hematopoietic stem cells, and ABCB1 (ATP-binding cassette sub-family B member 1), a protein expressed in human fetal neural stem/progenitor cells at an early developmental stage [51]. Hepatocyte nuclear factor 1-alpha (HNF1) gene also had a fold-change index significantly higher compared to BM-MSCs. HNF1 is a transcriptional activator required for the expression of several human embryonic stem cell-specific genes involved in cell growth, cell adhesion, epithelial formation, immune system, and inflammation.

This evidence supports the idea that PO-MSCs have a distinct individual molecular profile that appears in part different from BM-MSCs. For example, POU2F1 and TFAP4 genes’ transcriptional regulators involved in cancer stem cells and cell cycle were upregulated compared to BM-MSCs [50].

In contrast, PO-MSCs showed a reduced expression of cytokines and growth factors (GDF6, KDR, FGF10, and GDF5) when compared to BM-MSCs [50]. Despite fact that PO-MSCs share many important characteristics with BM-MSCs, including the cellular phenotype and the multi-lineage potential, they also show different immune regulatory profiles. Immune-associated molecules (CD117, HLA-DR, PDL-1, and PDL-2) are lost in PO-MSCs, resulting in a reduction of immunoregulatory abilities such as the inhibition of lymphocyte proliferation and the regulatory T cell expansion [50].

Gene expression of the transcription factors T-bet, GATA3, RORC, and FOXP3 were evaluated in a set of 14 CRSwNP and 8 CRSsNP samples [52], which revealed that eosinophilic CRSwNP was characterized by a higher level of GATA3 gene expression compared to non-eosinophilic CRSwNP. Tbet, GATA3, RORC were higher in CRSsNP than CRSwNP, whereas there was little difference for the FOXP3 gene. The expression of RORC implicates an involvement of the nasal immune response [52] better preserved in the CRSsNP patients than those with polyps.

Next generation sequencing (NGS) [53] has been used to compare CRSwNP and controls using a bioinformatics approach based on data from Plager et al. [54] and Stankovic et al. [55]. NGS profiling represents a non-biased methodology to identify gene and pathway changes. The analysis gave a total of 538 Differential Expressed Genes (326 up-regulated and 212 down-regulated) with enrichment for hematopoietic cell lineage and salivary secretion pathways. Modules were also identified, which were highly associated with chemokine signaling pathways, Th1 and Th2 cell differentiation.

CRSwNP compared to normal control nasal tissue samples were used to obtain transcriptome profiles of mRNAs and long non-coding RNAs (lncRNAs) [56,57]. Following this, 265 differentially expressed lncRNAs and 994 mRNAs were identified, mostly associated with signal transduction. Enriched pathways included cytokine–cytokine receptor interactions and cell adhesion molecules. lncRNAs were identified, which regulate chemokine (C-C motif) ligand 18 (CCL18), inflammation and polypeptide N-acetylgalactosaminyltransferase 7 (GALNT7) for cell proliferation. These genomic data provide a foundation for future investigations into mRNAs and lncRNAs as diagnostic and therapeutic targets in CRSwNP.

### 3.1. Characteristics of Nasal Polyp-Derived Mesenchymal Stem Cells

Mesenchymal stem cells are multipotent stromal cells that are present in multiple tissues, including bone marrow, fat tissue, and umbilical cord. MSCs are able to self-renew and have the potential to differentiate into adipocytes, osteoblasts, and chondrocytes in vivo and in vitro [58,59]. Under specific culture conditions, MSCs can differentiate into non-mesodermal lineages such as hepatocytes, neurons, astrocytes, pancreatic cells, cardiac muscle cells, or myocytes [60]. The MSCs can be obtained from several adult and fetal tissues, and do not carry ethical concerns such as embryonic stem cells (ESCs), but are more limited in terms of expansion and differentiation capabilities.

Nasal polyp tissue has been explored as a novel source of MSCs maintaining the stemness features and differentiation potential following multiple rounds of passaging [29], these Nasal polyp-derived MSCs (PO-MSCs) show a spindle-shaped morphology and typical features of MSCs [61,62,63]. The PO-MSCs phenotype is similar to that of MSCs derived from bone marrow or adipose tissue and is characterized by a positive expression for classical mesenchymal surface antigens, CD105, CD44, CD54, CD90, and CD73 [29,50,62], and a negative expression for hematopoietic surface markers (CD34, CD45, and HLA-DR). The PO-MSCs show high clonogenicity ability and can be passaged up to 15 times, maintaining their self-renewal ability [62]. These PO-MSCs are adult multipotent stromal somatic stem cells, able to differentiate into the classical mesenchymal-derived cell types, osteocytes, adipocytes, and chondrocytes, as well as having the ability with the appropriate stimulus to form neuron-like cells [50,62,64,65].

### 3.2. Osteogenic Differentiation

Initially, PO-MSCs have a fibroblastoid appearance, after osteogenic induction take on a cuboidal shape and the deposition of calcium salt nodules appears [62]. Osteogenic lineage commitment is supported by the expression of osteoblast-specific genes as RUNX2, the osteogenic master regulator, and osteocalcin, a late marker for osteoblastic maturation [66,67].

MSCs obtained from hypertrophied and contralateral normal inferior turbinate tissues derived from patients without CRS undergoing nasal surgery showed the similar differentiation potential, with increased osteoblast-specific gene expression such as BSP, RUNX2, BMP2, OSX, and COL1 [68].

### 3.3. Adipocyte Differentiation

When the PO-MSCs are grown in an adipogenic induction medium for 21 days, some cells showed a tendency to form spherical accumulations of multiple intra-cellular lipid-filled droplets [50,62], which can be detected by Oil Red O staining. These PO-MSCs express an increased gene expression of the PPARγ, a key player in controlling the transcriptional pathway of adipogenesis, as well as the target gene FABP4 [57] and the transcription factor ZNF423 as found in adipocytes derived from mesenchymal stem cells [69,70]. In addition, recent evidence suggests that the fine balance between transcription zinc finger proteins, such as ZNF521/ZNF423, is relevant for maintenance of stemness in mesenchymal stem and progenitor cells [69,71,72].

### 3.4. Chondrocyte Differentiation

PO-MSCs are also able to generate chondrocyte-like cells in vitro PO-MSCs induced with the chondrogenic medium that acquired a rounded and enlarged morphology and expressed the chondrogenic differentiation markers Sox9 and Col2A [62,73]. Sox9 is a transcription factor involved in cartilage formation and exerts its function as activator of type II collagen, the main component of cartilage [74].

### 3.5. Neural Differentiation

PO-MSCs can differentiate in vitro into cells of non-mesodermal origin, such as neuron-like cells. Cho et al., [62] displayed differentiated human neural progenitor cells expressing specific neural markers such as the neurofilament heavy chain (NF-H) and *neurofilament light chain (NF-L),* as well as a significant positivity for TuJ1 and decreased fibronectin expression. In addition, when the PO-MSCs were cultured as xenogeneic co-cultures with sliced adult rat brain biopsies neurofilament, nestin and GM-CSF could be detected [64]. PO-MSCs showed the same trans-differentiation ability when they are isolated from inferior turbinate as well as olfactory tissues [50]. Delorme and Girard [65,75] showed that olfactory ectomesenchymal stem cells (OE-MSCs), which originate from a neural crest-derived tissue could differentiate towards osteocytes as well as neuronal-like cells, when stimulated for neural differentiation, showed an increased expression of neural cell-related proteins including β-tubulin III, Nestin, GFAP, O4, and MAP2.

Table 2 summarizes master markers expressed during PO-MSCs’ multi-lineages differentiation.

The PO-MSCs cells had a relative disinclination to give rise to chondrocytes or adipocytes compared to classical MSCs sources, ADSCs, ASCc and BMSc. Systematic studies are required to determine the relative ability of nasal polyps to form MSCs and differentiate into the different types of cells compared to normal nasal tissue from different parts of the nose as either healthy adjacent biopsies or control normal subjects. Comparisons with other sources of MSCs from the bone marrow, adipose tissue and umbilical cord would be useful to appreciate the relative abilities for each cellular lineage. Different MCSs will have an inherent complement of suppressing and activating transcription factors, which will determine the degree of response for each type of differentiation stimulus.

Presently, there is considerable interest in the characterization of MSCs isolated from different sources, including those of the nasal tissues and polyps, which could be explored given the potentiality regenerative medicine. Experiments have been performed [76] where BM-MSCs were co-cultured with nasal polyp-derived cells cultures that exhibited a direct immunomodulation on nasal inflammatory polyposis that resulted in a significant increase in CD4^+^CD25^+^Foxp3^+^ T cells and a decrease in the frequency of CD4^+^, CD8^+^, CD14^+^, and NK cells, and finally promoted a strong inhibition of CD4^+^ and CD8^+^ T cell proliferation [76], where the global cytokine profile changed from an inflammatory to an anti-inflammatory response.

In other systems, co-culture experiments [77] on mouse ASCs exerted an immunomodulatory effect in regulating the allergic airway diseases in eosinophilic infiltrations consisting in a down-regulation of Th2 cytokines and in an up-regulation of Th1 and regulatory cytokines. In models of chronic kidney disease, hMSCs have shown by injection in mice to have a significant antifibrotic effect in preventing the renal injury by reducing the markers of fibrosis (e.g., collagen, TGF-β, and α-SMA) and increasing the production of protective molecules (e.g., HGF, E-cadherin, and BMP-7) [78].

Mesenchymal stem cells and those derived from nasal tissues, despite being a candidates for the treatment of degenerative/inflammatory diseases, as cells or as indirectly using co-culture medium, have many problems related to their use; manipulation in the laboratory, is complex and delicate to ensure the vitality and to retain stemness of the expanded cells, to the decreased regenerative capacity of the same cells to trigger a regenerative process once they are removed from their microenvironment and intrinsic variability in response stimuli between patients as well as that of compatibility can emerge.

## 4. Conclusions

**Figure 1:**
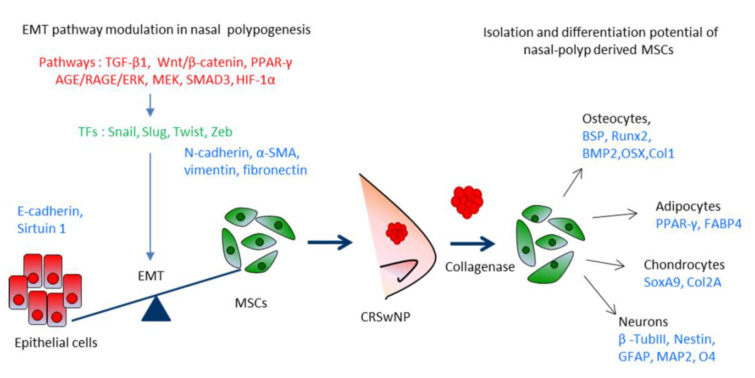
From nasal polyp biogenesis with EMT to MSCs isolation. Epithelial cells from the mucosa of the CRSsNP express specifically high levels of E-cadherin and can undergo EMT in response to a series of factors/pathways (TGF-β1, AGE/RAGE/ERK/MEK, SMAD/HIF-1α, Wnt/β-catenin, and PPARγ), which induce transcription factors (TFs) typically required for EMT remodeling (Snail, Slug, Twist, and Zeb). This process results in the formation of mesenchymal cells as an important component of the nasal polyps (CRSwNP). Biopsies of polyps can be disaggregated and MSCs selected, cultivated in vitro, and induced to differentiate into osteocytes, adipocytes, chondrocytes, and neuron-like cells, where characteristic proteins are detected.

Chronic rhinosinusitis with nasal polyps (CRSwNP) is one of the most common respiratory diseases worldwide. This disorder affects over ten percent of the adult population and the prevalence increases with age, causing a significant reduction in patients’ quality of life. Although the molecular pathogenesis of CRSwNP is not completely clear, EMT has been identified to play a role in the nasal tissue remodeling together with the persistent inflammatory environment. At present, the treatment options for CRS include the use of oral antihistamines to relieve symptoms of allergies, antibiotics to cure the chronic or recurring infection, and topical steroids to reduce the inflammation. The recurrence of polyps and symptoms occurs very frequently in patients with CRSwNP even after pharmacological and surgical treatment. There have now been developed humanized antibodies targeting IL-4/13 and IL-5 or their receptors expressed on eosinophils and basophils, which are able to exert a potent neutralizing activity. Even so, investigations into the signaling pathways associated with the formation of polyps can be fundamental to understating inhibitors that could be crucial in identifying new therapeutic targets. In addition, nasal polyps represent an alternative source of MSCs (PO-MSCs), having similar features found in BM-MSCs. Nasal polyp-derived mesenchymal stem/progenitor cells are an amenable model for in vitro investigation for molecular mechanisms underlying the inflammatory process responsible of nasal tissue remodeling, as delineated in Figure 1. The PO-MSCs, because of their immunomodulatory properties, could represent a promising treatment for several human diseases and in the future be used for the development of regenerative therapies. The development of suitable animal models for CRSwNP will aid this approach.

## Figures and Tables

**Table 1:** Main signaling pathways involved in controlling the initiation and progression of epithelial to mesenchymal transition (EMT) during neural polypogenesis. The formation of the nasal polyp is supported by a marked tissue remodeling process induced by various inflammatory mediators and modulation of intra-cellular signaling pathways. The resulting EMT can be inhibited by signal transduction inhibitors, leading to the loss of epithelial gene expression and the simultaneous acquisition of the molecular component properties typical of mesenchymal cells.

PATHWAYS	CRSwNP	Inhibitors of EMT	Ref.
TGF-β1	downregulated	HDAC1/2, TSA Chemical chaperones, PBA, PPB TGF-β1 miR-21 Glucocorticoids	[35,36,38,39]
SMAD3 and HIF-1α	upregulated	HIF-1α inhibitors 2ME2, 17-AAG	[40]
AGE/RAGE/ERK	upregulated	p38, MEK, ERK inhibitors	[41]
MEK1/2-ERK1/2	upregulated	MEK, ERK inhibitors	[42,43,44]
WNT/ β-catenin/GSK	upregulated	Wnt inhibitors IWP2 oxide 1,8-cineol inhibitor GSK-3	[45,46,47,48]
PPAR-γ	upregulated	agonist PPAR-γ Rosiglitazone	[49]

**Table 2:** Biomarkers identified after differentiation stimuli from mesenchymal stem/progenitor cells (MSCs) derived from nasal tissue or nasal polyps. Polyp derived mesenchymal stem cells (PO-MSCs) can differentiate, under specific stimuli, into adipocytes, osteocytes, chondrocytes, and neuron-like cells. The terminal differentiation of PO-MSCs towards cells of mesodermal or non-mesodermal origin results in the expression of lineage-specific markers.

Differentiation	Markers	Ref.
Adipogenesis	PPARγ, FABP4,	[50,62]
Osteogenesis	Runx2, Osteocalcin	[62]
Chondrogenesis	Sox9, Col2A	[62]
Neuron-like cells	NF-H, NF-L, TuJ1, Nestin, GM-CSF	[62,64,65,75]

## Abbreviations

CRS	Chronic rhinosinusitis
EPOS	European Position Paper on Rhinosinusitus and Nasal Polyps
CRSwNP	CRS with nasal polyps
CRSsNP	CRS without nasal polyps
AFRS	allergic fungal rhinosinusitis
eCRS	eosinophilic CRS
CCAD	central compartment allergic disease
GPA	Granulomatosis with polypangiitis
PO-MSCs	Polyp derived mesenchymal stem cells
EMT	Epithelial-mesenchymal transition
α-SMA	alpha-smooth muscle actin
MMP	Matrix metalloproteinases
TGF-β	Transforming growth factor beta
SMAD	Small Mothers Against Decapentaplegic
IFNγ	Interferon γ
Th2 T helper	Type2 T helper
ILC2	Type2 innate helper lymphoid cells
CCL11	Eotaxin-1
HIF-1α	Hypoxia-inducible factor
2ME2	Methoxyestradiol
17-AAG	17-allylaminogeldanamycin
AGE	Advanced Glycation Endproducts
RAGE	Receptor for advanced glycation endproducts
ERK	Extracellular signal-regulated kinases
MEK	Mitogen-Activated Protein Kinase Kinase
Wnt	Wingless-Type MMTV Integration Site Family
GSK3	Glycogen synthase kinase 3
PPARγ	Peroxisome proliferator-activated receptor gamma
XBP1	X-box binding protein 1
PBA	4-phenylbutylic acid
PP2	c-Src kinase inhibitor
GRP78	Glucose-regulated protein 78
PNECs	Primary nasal epithelial cells
PTEN	Phosphatase And Tensin Homolog
MAPK	Mitogen-Activated Protein Kinase
NF-κB	Nuclear factor-κB
JAK-STAT	Janus Kinase-Signal transducer and activator of transcription
HNEpCS	Human Nasal Epithelial Cells
ROG	Rosiglitazone
ZO-1	Zonula occludens-1
HDAC	Histone Deacetylase
LPS	Lipopolysaccharide
PROM1	Prominin-1
ABCB1	ATP-binding cassette sub-family B member 1
HNF1	Hepatocyte nuclear factor 1-alpha
BM-MSCs	Bone marrow mesenchymal stem cells
POU2F1	POU Class 2 Homeobox 1
TFAP4	Transcription Factor AP-4
GDF6	Growth Differentiation Factor 6
KDR	Kinase Insert Domain Receptor
FGF10	Fibroblast Growth Factor 10
GDF5	Growth/differentiation factor 5
HLA-DR	Major Histocompatibility Complex, Class II, DR Alpha
PDL-1	Programmed death-ligand 1
PDL-2	Programmed death-ligand 2
RORC	RAR Related Orphan Receptor C
HMGB1	High mobility group box 1 protein
FOXP3 -2	Forkhead box protein 3-2
CCL18	C-C motif) ligand 18
GALNT7	N-acetylgalactosaminyltransferase 7
RUNX2	Runt-related transcription factor 2
BMP2	Bone morphogenetic protein-2
BSP	Bone sialoprotein
OSX	Osterix
COL1	type I collagen
FABP4	Fatty acid-binding protein 4
Sox9	SRY-Box Transcription Factor 9
Col2A	type IIA collagen
NF-H	Neurofilamen heavy chain
NF-L	Neurofilamen light chain
GFAP	Glial fibrillary acidic protein
MAP2	Mutual protection of microtubule-associated protein 2
O4	Oct4
ADSC	Adipose stem cell
